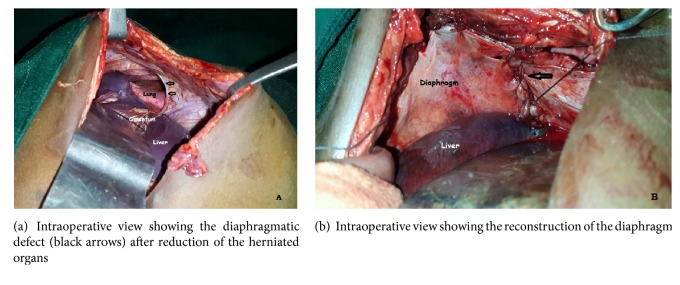# Isolated Right-Sided Posttraumatic Diaphragmatic Hernia

**DOI:** 10.1155/2018/8758021

**Published:** 2018-02-19

**Authors:** Ibrahim Amadou Magagi, Oumarou Habou, Harissou Adamou, Ousseini Adakal, Mahamoud Omid Ali Ada, Hellé Moustapha, Habibou Abarchi

**Affiliations:** ^1^General Surgery Department, National Hospital of Zinder, Zinder, Niger; ^2^Pediatric Surgery Department, National Hospital of Zinder, Zinder, Niger; ^3^Faculty of Health Sciences, University of Zinder, Zinder, Niger; ^4^General Surgery Department, Regional Hospital of Maradi, Maradi, Niger; ^5^Faculty of Health Sciences, University Dan Dicko Dankoulodo, Maradi, Niger; ^6^Pediatric Surgery Department, National Hospital of Lamordé, Niamey, Niger; ^7^Faculty of Health Sciences, Universite Abdou Moumouni, Niamey, Niger

## Abstract

Diaphragm is a compliant musculoaponeurotic barrier located between thoracic and abdominal cavities. Traumatic diaphragmatic rupture is a rare clinicopathological entity. We report a case of right-sided posttraumatic hernia in a child following blunt trauma to highlight diagnostic difficulties and therapeutic specific aspects. A 10-year-old boy was admitted to the emergency surgical department with thoracic trauma following pedestrian accident. At admission a haemothorax was suspected and treated by pleural drainage. The diagnosis of a right-sided diaphragmatic rupture was made after computed tomographic scan forty-eight hours later. At surgery, a reduction of herniated abdominal content and a suture of diaphragmatic defect were performed. The postoperative recoveries were uneventful and the patient was followed up for 12 months without symptoms. The possibility of a diaphragmatic rupture should be kept in mind and sought after any trauma of the thoracoabdominal junction as the diagnosis can be challenging in emergency department.

## 1. Introduction

The diaphragm is a dome-shaped and thin musculoaponeurotic barrier that plays an important role in respiratory function [1, 2]. This location, between chest and abdomen, exposes it to injury by closed or penetrating trauma to the thoracoabdominal transition area on either side [1, 2]. Traumatic diaphragmatic rupture is a rare clinicopathological entity [1, 2]. It occurs after severe blunt or penetrating traumas [3]. Right-sided diaphragmatic rupture is less common than the left-side, as a result of the protective anatomic lie of the liver [2, 3]. The diagnosis of a right diaphragmatic injury is a challenge for the surgeon because clinical signs are often nonspecific [1, 2]. Only half of cases are diagnosed early and this fact may lead to progressive herniation of intra-abdominal contents into the thorax [1]. Once diagnosed, the treatment is performed by conventional or laparoscopic surgery [1–3]. We report a case of right-sided posttraumatic hernia in a child following blunt trauma to highlight the diagnostic difficulties and the therapeutic peculiarities.

## 2. Case Presentation

A 10-year-old boy was admitted for thoracic trauma following a pedestrian accident. At admission, the patient was conscious and hemodynamically stable, with mild dyspnea. The clinical examination showed some straight small abrasions over the right side of the chest; no other external injuries were apparent. The right hemithorax was dull to percussion and the vesicular murmur was abolished. The rest of the examination was unremarkable.

**Figure 1:**
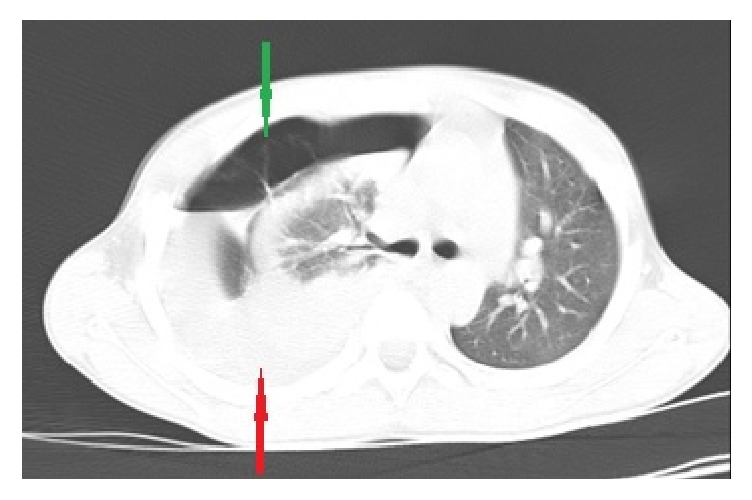
Thoracic CT scan showing the presence of digestive loops (green arrow), and right lobe of the liver (red arrow) with compression of the right lung and shift of the mediastinum to the left.

The thoracic X-ray showed an opacity of the right pulmonary field, evocative of an effusion of great abundance, without any fracture of the ribs. The biological balance revealed anemia at 8 g/dl. The diagnosis of haemothorax was made. Drainage of the right hemithorax was performed and allowed the extraction of 200 cc of blood. Forty-eight hours later, no clinical improvement was observed, with persistence of dyspnea. Thoracic and abdominal computed tomographic scan (CT scan) was requested and showed herniation of the right lobe of the liver along with bowel loops into the right hemithorax (Figure 1). A rupture of the right diaphragmatic dome was confirmed.

The surgical treatment was performed by a right subcostal laparotomy. Exploration found an intrathoracic ascent of the right lobe of the liver, transverse colon, and omentum. Anterior diaphragmatic disinsertion of about 15 cm was found with an L-shaped recess extending about 5 cm posteriorly (Figure 2(a)). The herniated organs were reintroduced into the abdominal cavity. A pleural drainage and a primary repair of the diaphragmatic defect with nonabsorbable suture were made (Figure 2(b)).

Postoperative recovery was uneventful and the patient was discharged on day 10. The patient was followed up for 12 months without any symptoms.

## 3. Discussion 

Diaphragmatic ruptures are uncommon, especially in children, occurring in 0.8–7% of blunt trauma patients and 10–15% of penetrating trauma patients [3–8]. Traumatic diaphragmatic injury is often a hidden evidence of serious injury to thoracic or abdominal viscera [2, 8]. The diaphragmatic rupture would be the result of a transmission of intense forces between the abdominal and thoracic compartments via the viscera or an abrupt increase in intra-abdominal pressure [6, 7].

The left hemidiaphragm would appear to be more often affected than the right side due to the protective effect of the liver [1, 3–5]. Petrone et al. [1] reported a traumatic diaphragmatic hernia rate of 75% on the left versus only 25% on the right side. However, an increase of incidence on the right side is reported due to an improvement in imaging techniques. The relative flexibility of the suspensory ligament of the liver in the child offers less protection to the right side [6]. But many authors believe that the incidence of right diaphragmatic traumatic ruptures is underestimated. Thus, autopsy series demonstrated that there was an equal incidence between right and left lesions, leading some authors to wonder whether the left diaphragmatic hernias were more frequent or simply easier to diagnose [1, 5]. Indeed, a right traumatic diaphragmatic rupture can remain unnoticed for weeks or even years [3].

Diaphragmatic ruptures were classified by Grimes in 1974, in 3 stages according to the presentation time: acute, which are diagnosed at the time of injury; delayed, presenting after an interval of time since the original injury; and chronic, presenting only with symptoms of visceral incarceration due to the associated herniation of abdominal contents into the thorax [3, 7, 8]. In our case, the rupture of the right diaphragmatic dome probably occurred concomitantly with the accident, but the hernia was initially taken for an isolated hemothorax.

The main cause of traumatic diaphragmatic rupture was blunt trauma following traffic accident and falls from heights [3, 5].

Clinically, in the acute phase, the symptomatology is thoracoabdominal with lumbar or epigastric pain, decreased vesicular murmur in the right pulmonary base, dyspnea, right hemothorax, right shoulder pain, and, more rarely, at auscultation, a thoracic peristalsis [3, 7]. When the defect is small and there is no additional major organ injury, the diagnosis is often missed, and the patient may present respiratory symptoms or intestinal obstruction in the following days [3, 7].

X-ray and CT scan are most commonly used techniques for diaphragmatic rupture diagnosis [3, 4]. The chest radiography can be an important tool for life-threatening injuries identification (hemo- or pneumothorax) or diaphragm injuries in the evaluation of trauma patients [2, 4]. The chest X-ray could be normal or show marked elevation of one hemidiaphragm, especially on the right side [4, 5], thus giving low sensitivity to this examination. Helical CT scan has better sensitivity to diagnose right diaphragmatic rupture, by showing discontinuity of the hemidiaphragm, the dependent viscera sign, the collar sign, and intrathoracic herniation of abdominal contents [7, 8]. Ultrasound, magnetic resonance imaging, and upper gastrointestinal contrast study could be also useful [1, 6].

Surgical treatment may be realized either through laparotomy or thoracotomy or video-assisted approach [1, 3]. Its objective is the reduction of the hernial content, pleural drainage, and repair of the defect [7]. Laparotomy is more appropriate in unstable patients when associated intra-abdominal injuries are suspected [2, 3, 6]. Thoracotomy is necessary to handle late diaphragmatic hernia and isolated lesions of the right diaphragm and in case of expected chest injury [2, 3]. The repair of the small diaphragmatic defect is usually done with nonabsorbable suture material and rarely with slowly absorbable stitches [3, 7]. Prosthetic mesh is required in large defect to provide tension-free repair [3].

Mortality and morbidity in right diaphragmatic rupture are often due to associated intra-abdominal or intrathoracic injuries. Mortality is almost nil in isolated diaphragmatic rupture [3, 5].

## 4. Conclusion 

Right diaphragmatic rupture is a rare condition especially in children. The diagnosis is difficult and often delayed. The management is surgical. Prognosis is related to associated injuries. The possibility of a diaphragmatic rupture should be kept in mind and sought after any trauma of the thoracoabdominal junction as the diagnosis can be challenging in emergency department.

## Figures and Tables

**Figure 2:**